# Social capital, digital economy and the entrepreneurship of the new generation of migrant youth: Empirical analysis based on CMDS data

**DOI:** 10.1371/journal.pone.0322458

**Published:** 2025-06-25

**Authors:** Jinmei Wang, Xiangshu Dong, Jiaan Yang

**Affiliations:** 1 School of Economics and Management, Tianjin Chengjian University, Tianjin, China; 2 School of Economics, Capital University of Economics and Business, Beijing, China; Universidad Autónoma de Baja California: Universidad Autonoma de Baja California, MEXICO

## Abstract

In the era of digital economy, the form of digital production promotes the transformation of enterprise organization and business operation mechanism. It also affects the development of regional entrepreneurship and the choice of individual entrepreneurship. Youth is the future of the country and the hope of the nation, and youth employment is related to people’s well-being, economic development and the future of the country. From the perspective of social capital, this paper examines the entrepreneurship of the new generation of migrant youth by using the dynamic monitoring data of the floating population in 2018(CMDS). The empirical results show that the improvement of social capital can significantly promote the entrepreneurship of the new generation of migrant youth; From the perspective of social capital, social network, social trust and social status promote entrepreneurship significantly. However, the role of social participation is not obvious. From the perspective of different types of entrepreneurship, compared with survival entrepreneurship, social capital has a more significant impact on opportunity entrepreneurship. Heterogeneity analysis found that, Social capital has a significant impact on opportunistic entrepreneurship of groups with higher education level, higher family income and modern service industry; In the central cities and coastal areas, social capital can promote the opportunity entrepreneurship of the new generation of migrant youth. Expansion analysis further found that the digital economy can significantly promote the entrepreneurial effect of social capital.

## 1. Introduction

Entrepreneurship is the source of power to promote sustainable economic growth, and young people are more active in entrepreneurial activities [1]. General Secretary Xi Jinping emphasized that “the whole society should attach importance to and support young people’s innovation and entrepreneurship, and provide more favorable conditions. “Build a broader stage, so that the majority of young people in innovation and entrepreneurship glow with more dazzling youth [2]. In 2015, China’s government work report clearly put forward “mass entrepreneurship and innovation” [3]. In 2017, the State promulgated the Medium and Long-term Youth Development Plan (2016–2025), which listed youth employment and entrepreneurship as one of the ten major areas of development. Stimulate the vitality of youth innovation and entrepreneurship. With the continuous improvement of China’s domestic market and the increasingly frequent flow of labor force, young people are the main force of social entrepreneurship. The new generation of migrant youth is an important part of the youth entrepreneurship group in China. Although the new generation of migrant youth has a strong spirit of adventure and innovation [4], they also have less experience and younger age. Instability, low growth potential and high failure rate of entrepreneurship [5]. Under the background of high employment pressure, how to change passive employment into active entrepreneurship? Stimulating the entrepreneurial vitality of the new generation of young migrant workers has become an urgent social and economic problem in China.

Social capital plays an important role in entrepreneurial activities [6]. Many studies believe that the more social capital you have, the more conducive it is to entrepreneurship [7,8]. Although there have been some discussions on social capital and entrepreneurship, However, the relevant research on the new generation of migrant youth, a special group, needs to be deepened. Under the background of digital economy, digital technology represented by artificial intelligence, Internet of Things and big data participates in entrepreneurship activities. It breaks through the traditional business model and becomes a new lever and new momentum for entrepreneurship. Then, how will social capital affect the entrepreneurial decision-making and entrepreneurial types of the new generation of migrant youth? What role do the dimensions of social capital play in the entrepreneurship of the new generation of migrant youth? What is the heterogeneous impact of social capital on the entrepreneurship of the new generation of migrant youth in different groups? What role does the digital economy play in this impact? These problems need to be further studied in combination with data. This paper intends to discuss these issues and provide empirical evidence on how to improve the entrepreneurial level of the new generation of migrant youth in the future.

## 2. Theoretical analysis and research hypothesis

### 2.1. Research on social capital and entrepreneurial decision-making

The network of interpersonal relationships in social development is called social capital. Social capital mainly covers trust, norms and networks within society, and improves the efficiency of social operation through coordinated action [9]. At present, social capital is regarded as the third form of capital after material capital and human capital. It has become an important perspective for entrepreneurship research [10]. Firstly, social capital is conducive to information exchange and provides more entrepreneurial opportunities for young entrepreneurs [11]. Entrepreneurial activities require high information, and young migrant workers leave their hometown with relatively little information about entrepreneurship. Through social interaction, young people who have left their homes can enhance the exchange of relevant information and obtain more entrepreneurial information and knowledge [12,13]. Second, social capital is an informal norm with universal trust as its core [14,15]. Higher social capital is conducive to mutual trust among network members, promoting cooperation and sharing among entrepreneurs, and alleviating information asymmetry. Reduce the cost and risk of entrepreneurship [16–18]. Social trust can also directly affect formal institutions (Putnam, 1993) [19] by reducing transaction costs and opportunism [20,21], stimulate economic vitality. Especially for young entrepreneurs, social capital is an important “adhesive” for resource integration. When social capital is high, they can cooperate better with social network members and share entrepreneurial risks. Thirdly, the entrepreneurial process often has a high demand for capital, and in the context of large corporate financing constraints as well as the development of the financial market that is still to be improved, young mobile entrepreneurs with high social capital can borrow through social networks to broaden the channels of capital and reduce the cost of financing [22]. To sum up, this paper puts forward the following assumptions:

Hypothesis 1: Social capital is conducive to the entrepreneurship of young migrants.

### 2.2. Research on social capital and types of entrepreneurship

At present, there is no uniform standard for the classification of entrepreneurship types in academia. According to different entrepreneurial motivations, the Global Entrepreneurship Monitor (GEM) first defined opportunity entrepreneurship and survival entrepreneurship in 2001 [23]. Opportunistic entrepreneurship is a kind of entrepreneurship that individuals perceive business opportunities and take the initiative to start. Opportunistic entrepreneurship requires high technical level, access threshold and start-up capital of entrepreneurs. Survival entrepreneurship refers to the entrepreneurial choice that is forced to solve the basic livelihood problems. Its overall requirements for technical level, access threshold and start-up funds are low. For the young floating population, the opportunity entrepreneurship is more difficult, but the opportunity entrepreneurship brings more benefits. Opportunity entrepreneurship is also a key force to promote the development of modern economy. Higher social capital brings diversified contacts, which is more conducive to entrepreneurs to achieve opportunistic entrepreneurship [24]. Social capital also enables entrepreneurs to obtain more entrepreneurial information and financial support. The incentive effect on opportunistic entrepreneurship is more significant [25]. To sum up, this paper puts forward the following preliminary assumptions:

Hypothesis 2: Social capital is more conducive to opportunity entrepreneurship than survival entrepreneurship.

### 2.3. Social capital, digital economy and entrepreneurial decision-making

The rapid development of digital economy provides new opportunities for entrepreneurship. Social capital and information resources have an important impact on entrepreneurship, and the digital economy, which as the carrier of modern information networks, promotes the widespread application of digital technology, provides a new path for social capital, is conducive to make up for the constraints of the lack of individual social resources, eases the information asymmetry in the process of entrepreneurship, compensates for the risk of entrepreneurial failure due to the lack of social capital of young people, and promotes entrepreneurship [26]. On the one hand, the digital economy can break the space-time distance barrier of the dissemination of knowledge and innovation elements, and improve the mobility of entrepreneurship elements. Release the vitality of multiple needs, help to establish virtual trading places, and provide a new social network platform for teenagers. It speeds up the dissemination of knowledge and information, and provides more entrepreneurial opportunities and digital information. On the other hand, the digital economy has changed the traditional mode of entrepreneurship, and new industries and new formats have emerged. It activates the labor market, reduces the cost of communication to a great extent, and stimulates the enthusiasm and motivation of young people to start their own businesses. Promote the young floating population to seize the opportunity of the times to start their own businesses. In addition, the digital economy has a strong social interactivity, which to a certain extent broadens the depth and breadth of the social network process and accumulates social capital for teenage entrepreneurship, which are all conducive to the development of entrepreneurial activities. To sum up, this paper puts forward the following assumptions:

Hypothesis 3: The digital economy plays a positive facilitator in social capital impacting entrepreneurship.

## 3. Research design

### 3.1. Data source and processing

The research object of this paper is the new generation of migrant youth, considering the existing research [27], the sample aged 16–34 years old is retained. In order to ensure the reliability of the results, the original data are processed as follows: Delete those who have missing values, whose employment status is other, and who have never worked for more than one hour with income in the week before May Day in the survey year. Work, including family or self-employment, and samples with personal income and family income less than or equal to 0; Secondly, we only consider the floating population who go out to work, work and do business. The reasons for mobility are other, birth, marriage, family migration, pension in different places, relatives and friends, demolition and relocation, and self-care. For the floating population who take care of their children and the elderly, their entrepreneurship and employment probability rarely occur. Therefore, this kind of sample should be rejected to reduce the sample self-selection bias to a certain extent. Finally, 38808 valid baseline observations are obtained. The main sources of data used in this paper are as follows: social capital and entrepreneurship data from China’s Migrant Population Dynamic Monitoring Survey Data (CMDS) published by the National Health and Family Planning Commission in 2018, digital economy data from the “China Urban Statistical Yearbook” and “the Digital Inclusive Finance Index”.

### 3.2. Variable selection

#### 3.2.1. Explained variable.

Entery: According to the classification of employment status by the International Labour Organization and the United Nations [28], Divide the answers to “What is your current employment status” in the existing sample questionnaire into two categories. Among them, the samples who answered “employer” and “self-employed worker” were identified as entrepreneurs and assigned a value of 1. The samples that answered “employees with fixed employers” and “employees without fixed employers (part-time workers, casual workers, etc.)” were identified as others. Assign 0.

Types of entrepreneurship: Considering the different motivations for entrepreneurship, according to the Global Entrepreneurship Monitor (GEM) and the existing research on the classification of entrepreneurship [29,30]. This paper further classifies the group of entrepreneurs who answered “employer” was classified as “opportunistic entrepreneurship” (Oppty), with a value of 1 and others of 0;The group that answered “self-employed workers” was classified as “survival entrepreneurship” (Surv) and assigned a value of 1, while the others were 0. Opportunistic entrepreneurship is where entrepreneurs proactively engage in entrepreneurial activities in order to pursue entrepreneurial opportunities and achieve their personal goals, and these individuals usually have a high tolerance for risk. Survival entrepreneurship is where entrepreneurs are forced to start a business for basic subsistence, and they may be limited in the utilization of resources.

#### 3.2.2. Explanatory variables.

The core explanatory variable of this paper is social capital (SC), which refers to the sum of resources embedded in the social network of a person’s status and relationship network in the social structure, and is a combination of network, trust, status and participation, and is examined in this paper from the four dimensions of Social network (SI), social trust (ST), social status (SEI) and social participation (SA) [31]. Social network is a kind of interactive relationship agreed upon in interpersonal communication, reflecting the size of the interpersonal circle and the strength of resources obtained, and select “whether to participate in the alumni association, hometown association, hometown chamber of commerce organization” as the proxy variable of the social network [32], one represents participation and zero means no participation. Social trust has an important impact on human behavioural decision making, reflecting the level of trust in each other, and select “Whether to agree with local people to accept themselves as a member” and “whether local people look up to outsiders” as proxy variables of social trust. Social status is a comprehensive indicator of the social relations established by an individual or organization through prestige, etc., symbolizing relations of authority in the process of interaction, calculated on the basis of the level of education, personal income and occupation [33]. Social participation reflects the self-expression of individual rights, and select “Whether to participate in the activities of Party/League organizations, participate in Party branch meetings”, “whether to comment on state affairs and social events on the Internet, Participation in discussion “as a proxy variable of social participation, these two indicators use 1-4 scoring method, the higher the score, the more positive it is.”. In this paper, the principal component analysis was carried out after standardization of each index, and the KMO value passed the test. The χ2 statistic value of Bartlett sphericity test reached a significant level (p = 0.000), indicating that it was suitable for factor analysis. Therefore, the common factor is regarded as the proxy index of social capital. At the same time, through factor analysis, the proxy variables of four dimensions of social capital (social network, social trust, social status, social participation) are constructed.

#### 3.2.3. Control variables.

In order to accurately measure the net effect of social capital, combined with the existing research, The variables that may affect the entrepreneurship of young migrants are controlled from three aspects: personal characteristics, family characteristics and mobility characteristics.

In terms of individual characteristics, ① Age is obtained by subtracting the year of birth from the year of sample survey. At the same time, the age square term (Age_sq) is introduced; ② Gender, 1 = male, 0 = female;③ Marry status, first marriage, married and widowed are classified as married, with a value of 1, and divorce, cohabitation and unmarried are classified as unmarried, with a value of 0;④ Educational attainment (Edu),The assigned values are not in primary school = 1, primary school = 2, junior high school = 3, senior high school/technical secondary school = 4, junior college = 5, undergraduate = 6, graduate = 7;⑤ Nation, 1 = Han, 0 = others; ⑥ Health, 1 = healthy, 0 = unhealthy;⑦ For health record (H _ record), assign the value of health record as 1, otherwise, it is 0;House property right of ⑧, 1 = self-owned property right, 0 = others; (9) Politic, assigned by CPC members as 1, otherwise as 0;⑩ Hukou, 1 = agricultural registered permanent residence, 0 = non-agricultural registered permanent residence.

Family characteristics: ① family income (F _ income). The logarithm of family monthly income reflects whether the start-up capital is sufficient [34]; ② Family size (F _ size). The number of families living together at the work site.

In terms of mobility characteristics, ① City _ num. The total number of cities that have left their hometown for the first time;② flow range (Fl _ range). There are three categories (1 = cross-county in the city, 2 = cross-city in the province, 3 = cross-province). In addition, provincial dummy variables are introduced to control the fixed effects of regions.

The results of descriptive statistics for all variables are shown in Table 1. In the total sample, there are 12694 entrepreneurs, accounting for 32.7%, of which 10607 are survival entrepreneurs and 2087 are opportunity entrepreneurs. They account for 83.6% and 16.4% of the total number of entrepreneurs respectively, which shows that survival entrepreneurship is still the main type of entrepreneurship among young migrants.

**Table 1:** Descriptive analysis.

Variable symbol	Variable name		Variable meaning	N	Mean	Std	Min	Max
Explained variable								
Entery	Entrepreneurial decision-making		1 = start a business; 0 = not start a business	38,808	0.327	0.469	0.000	1.000
Oppty	Opportunistic entrepreneurship		1 = Employer; 0 = Other	38,808	0.055	0.226	0.000	1.000
Surv	Survival entrepreneurship		1 = self-employed; 0 = other	38,808	0.273	0.446	0.000	1.000
Explanatory variable								
SC	Social capital	full sample	Composite index of social capital variables	38,808	0.000	0.606	−3.235	5.397
		High level of education		4,432	0.392	0.684	−0.596	5.377
		Low level of education		34,376	−0.051	0.576	−3.235	5.397
		High-income group		14,590	0.166	0.635	−2.452	5.377
		Low-income group		24,218	−0.100	0.565	−3.235	5.397
		Services industry		24,661	−0.007	0.612	−3.235	5.397
		Non-services industry		14,147	0.012	0.596	−2.680	5.377
		Modern Services industry		3,085	0.228	0.658	−0.950	5.374
		Traditional service industry		21,576	−0.040	0.598	−3.235	5.397
		Central cities		21,280	0.041	0.602	−2.680	5.397
		Outlying Cities		17,528	−0.049	0.608	−3.235	5.374
		Coastal areas		18,427	0.020	0.574	−2.452	5.377
		Inland areas		20,381	−0.017	0.633	−3.235	5.397
SI	Social Network		Composite index of social network variables	38,808	0.000	0.834	−0.448	3.962
ST	Social trust		Composite Index of Social Trust Variables	38,808	0.000	1.000	−4.130	1.515
SEI	Social status		The specific value is calculated according to education level, personal income and occupation.	38,808	−2.321	0.321	−5.898	0.168
SA	Social participation		Composite Index of Social Participation Variables	38,808	0.000	1.000	−0.307	10.499
Control Variables: Personal Characteristics								
Age	Age		Survey year minus year of birth (years)	38,808	28.880	3.662	16.000	34.000
Age_sq	Age squared		Age squared	38,808	8.475	2.037	2.560	11.560
Gender	Gender		1 = male, 0 = female	38,808	0.537	0.499	0.000	1.000
Marry	Marriage		1 = married; 0 = unmarried	38,808	0.702	0.457	0.000	1.000
Edu	Education		Level of education	38,808	3.911	1.121	1.000	7.000
Nation	Nation		1 = Han, 0 = Other	38,808	0.910	0.286	0.000	1.000
Health	Health		1 = healthy, 0 = unhealthy	38,808	0.997	0.051	0.000	1.000
H_record	Health records		1 = yes; 0 = no	38,808	0.354	0.478	0.000	1.000
House	Nature of housing		1 = own property; 0 = other	38,808	0.208	0.406	0.000	1.000
Politic	Political outlook		1 = CPC member; 0 = other	38,808	0.054	0.226	0.000	1.000
Hukou	Household registration		1 = Non-agricultural account; 0 = Agricultural account	38,808	0.167	0.373	0.000	1.000
Control Variables: Family Characteristics								
F_income	Household income		Logarithm of monthly household income (yuan)	38,808	8.760	0.582	4.605	11.513
F_size	Family size		Number of families living together at the work site (person)	38,808	2.832	1.260	1.000	10.000
Control variables: flow characteristic								
City_num	Number of mobile cities		Total number of mobile cities from the first time to the present (number)	38,808	1.947	1.452	1.000	40.000
Fl_range	Flow range		1 = cross county within city; 2 = cross city within province; 3 = cross province	38,808	2.319	0.742	1.000	3.000

### 3.3. Benchmark regression model

Because the explained variables of entrepreneurial decision and entrepreneurial choice of young migrant population in this paper are binary discrete variables, The binomial Logit model was used for estimation, and the benchmark regression model was as follows:



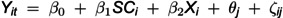

(1)


Where Y is the entrepreneurial decision or choice, I is the individual, and J is the province where the individual is located. SC is social capital and X is a series of control variables. θ is the regional fixed effect, and ζ is the random disturbance term, which is assumed to follow the logistic distribution. β1 is the parameter to be estimated.

## 4. Analysis of empirical results

### 4.1. Benchmark regression

#### 4.1.1. The impact of social capital on the entrepreneurial decision-making of young migrants.

Based on the econometric model (1), the impact of social capital on the entrepreneurial decision-making of young migrants is estimated, and the results are shown in Table 2. All regressions utilized the Logit binary regression model, and the regression coefficient results for all variables in the table represent marginal effects. After adding the control variable of individual characteristics in column (1), the estimated coefficient of social capital is significantly positive. After adding variables such as family characteristics and mobility characteristics in columns (2)- (3), the estimated coefficient of social capital is still significantly positive. It shows that social capital has significantly promoted the entrepreneurship of the new generation of mobile young people. The above empirical results support Hypothesis 1. This may be due to the fact that social capital provides a large number of social resources for youth entrepreneurship, increases the frequency of communication about youth entrepreneurship, reduces information opacity, and increases entrepreneurial opportunities.

**Table 2:** The estimated results of the influence of social capital on the entrepreneurial decision of young migrant population.

	(1)	(2)	(3)	(4)	(5)	(6)	(7)
	Entery	Entery	Entery	Entery	Entery	Entery	Entery
SC	0.057^***^	0.028^***^	0.028^***^				
	(0.004)	(0.004)	(0.004)				
SI				0.020^***^			
				(0.002)			
ST					0.011^***^		
					(0.002)		
SEI						0.264^***^	
						(0.011)	
SA							0.002
							(0.002)
Age	0.035^***^	0.035^***^	0.036^***^	0.036^***^	0.037^***^	0.032^***^	0.037^***^
	(0.009)	(0.009)	(0.009)	(0.009)	(0.009)	(0.009)	(0.009)
Age2	−0.048^***^	−0.051^***^	−0.053^***^	−0.053^***^	−0.054^***^	−0.047^***^	−0.054^***^
	(0.015)	(0.015)	(0.015)	(0.015)	(0.015)	(0.015)	(0.015)
Gender	0.005	0.009^**^	0.009^**^	0.011^**^	0.013^***^	−0.018^***^	0.013^***^
	(0.004)	(0.004)	(0.004)	(0.004)	(0.004)	(0.004)	(0.004)
Marry	0.209^***^	0.104^***^	0.103^***^	0.103^***^	0.103^***^	0.103^***^	0.103^***^
	(0.006)	(0.007)	(0.007)	(0.007)	(0.007)	(0.007)	(0.007)
Edu	−0.089^***^	−0.090^***^	−0.089^***^	−0.085^***^	−0.085^***^	−0.127^***^	−0.084^***^
	(0.002)	(0.002)	(0.002)	(0.002)	(0.002)	(0.003)	(0.002)
Nation	0.064^***^	0.055^***^	0.045^***^	0.046^***^	0.047^***^	0.038^***^	0.046^***^
	(0.008)	(0.008)	(0.008)	(0.008)	(0.008)	(0.008)	(0.008)
Health	0.034	0.022	0.018	0.019	0.017	−0.001	0.020
	(0.040)	(0.039)	(0.039)	(0.039)	(0.039)	(0.038)	(0.039)
H_record	−0.024^***^	−0.018^***^	−0.017^***^	−0.018^***^	−0.018^***^	−0.013^***^	−0.016^***^
	(0.005)	(0.005)	(0.005)	(0.005)	(0.005)	(0.005)	(0.005)
House	−0.029^***^	−0.056^***^	−0.049^***^	−0.049^***^	−0.051^***^	−0.049^***^	−0.049^***^
	(0.006)	(0.006)	(0.006)	(0.006)	(0.006)	(0.006)	(0.006)
Politic	−0.055^***^	−0.055^***^	−0.055^***^	−0.052^***^	−0.052^***^	−0.047^***^	−0.052^***^
	(0.012)	(0.011)	(0.011)	(0.011)	(0.011)	(0.011)	(0.011)
Hukou	−0.031^***^	−0.032^***^	−0.033^***^	−0.032^***^	−0.033^***^	−0.033^***^	−0.033^***^
	(0.007)	(0.007)	(0.007)	(0.007)	(0.007)	(0.007)	(0.007)
F_income		0.147^***^	0.143^***^	0.147^***^	0.149^***^	0.080^***^	0.150^***^
		(0.004)	(0.004)	(0.004)	(0.004)	(0.005)	(0.004)
F_size		0.030^***^	0.029^***^	0.029^***^	0.029^***^	0.036^***^	0.029^***^
		(0.002)	(0.002)	(0.002)	(0.002)	(0.002)	(0.002)
City_num			−0.003^**^	−0.003^**^	−0.002^*^	−0.003^**^	−0.003^*^
			(0.001)	(0.001)	(0.001)	(0.001)	(0.001)
Fl_range			0.038^***^	0.037^***^	0.039^***^	0.037^***^	0.038^***^
			(0.003)	(0.003)	(0.003)	(0.003)	(0.003)
Pro	Yes	Yes	Yes	Yes	Yes	Yes	Yes
N	38808	38808	38808	38808	38808	38808	38808

Note: Standard errors are in brackets.

*** ,

** and

* mean significant at 1%, 5% and 10% levels respectively

Furthermore, we also examine the impact of entrepreneurship from four dimensions of social capital. Through empirical research, this paper finds that social network, social trust and social status all significantly promote entrepreneurship of young migrants. However, social participation did not significantly increase the probability of entrepreneurship (see the estimated results in columns (4)-(7) of Table 2). The above results show that social network, social trust and social status are important dimensions of social capital. The difficulties of lack of opportunities and shortage of funds have effectively promoted the entrepreneurship of the young floating population. More developed social networks help young migrants to better share information and capture entrepreneurial opportunities. Higher social trust helps to reduce the transaction cost of entrepreneurship, and helps entrepreneurs to cooperate and share risks. Higher social status means that entrepreneurs have higher prestige and are in an advantageous position in social interaction. It is more conducive to gathering all kinds of entrepreneurial resources. Social participation is an important manifestation of social vitality and provides a better platform for entrepreneurship. However, from our empirical results, the impact of social participation of young migrants is limited. Limited by the lag of social construction, the channels of social and political participation of Chinese youth groups are not smooth at present. Especially, the young floating population who are outside the system may have the problem of “marginalization” of political participation. As a result, their enthusiasm for actual social participation is limited [35], Social participation also has a limited impact on the entrepreneurship of the new generation of mobile young people.

#### 4.1.2. The impact of social capital on the entrepreneurial types of young migrants.

To further study the impact of social capital on two different types of entrepreneurship, opportunity and survival, the estimated results are shown in Table 3. The estimation results of columns (1)-(3) show that social capital significantly increases the probability of opportunistic entrepreneurship of young migrants. For survival entrepreneurship, after controlling the characteristics of family and mobility, it is difficult to find a way to solve the problem. The effect of social capital is not significant (see columns (5)-(6)), thereby supporting Hypothesis 2. Compared with the passive survival entrepreneurial behavior, the young floating population has a strong motivation to realize their self-worth. Opportunistic entrepreneurship comes more from the internal motivation of entrepreneurs. Higher social capital will effectively reduce the cost of entrepreneurship, and will better stimulate the entrepreneurial vitality of young migrants. Compare columns (1)-(3) with column (5)-(7) in Table 4, It can be found that although social network, social trust and social status all significantly increase the probability of two kinds of entrepreneurship. However, compared with survival entrepreneurship, these three dimensions of social capital play a stronger role in promoting opportunistic entrepreneurship of young migrants. Social network, social trust and social status interact with each other to form a higher social capital. It has created good conditions for the opportunistic entrepreneurship of the young floating population. At the same time, our empirical results also show that the impact of social participation is not significant for both opportunity and survival types.

**Table 3:** Estimated results of the impact of social capital on the type of entrepreneurship of young migrants.

	(1)	(2)	(3)	(4)	(5)	(6)
	Oppty	Oppty	Oppty	Surv	Surv	Surv
SC	0.026^***^	0.014^***^	0.014^***^	0.020^***^	0.006	0.006
	(0.002)	(0.002)	(0.002)	(0.004)	(0.004)	(0.004)
Personal characteristics	Yes	Yes	Yes	Yes	Yes	Yes
Family characteristics	No	Yes	Yes	No	Yes	Yes
Flow characteristics	No	No	Yes	No	No	Yes
Pro	Yes	Yes	Yes	Yes	Yes	Yes
N	38808	38808	38808	38808	38808	38808

Note: Standard errors are in brackets.

*** ,

** and

* mean significant at 1%, 5% and 10% levels respectively

**Table 4:** The estimation results of the influence of each dimension of social capital on the type of entrepreneurship of young migrants.

	(1)	(2)	(3)	(4)	(5)	(6)	(7)	(8)
	Oppty	Oppty	Oppty	Oppty	Surv	Surv	Surv	Surv
SI	0.009^***^				0.006^***^			
	(0.001)				(0.002)			
ST		0.004^***^				0.006^***^		
		(0.001)				(0.002)		
SEI			0.088^***^				0.085^***^	
			(0.004)				(0.010)	
SA				0.001				0.001
				(0.001)				(0.002)
Personal characteristics	Yes	Yes	Yes	Yes	Yes	Yes	Yes	Yes
Family characteristics	Yes	Yes	Yes	Yes	Yes	Yes	Yes	Yes
Flow characteristics	Yes	Yes	Yes	Yes	Yes	Yes	Yes	Yes
Pro	Yes	Yes	Yes	Yes	Yes	Yes	Yes	Yes
N	38808	38808	38808	38808	38808	38808	38808	38808

Note: Standard errors are in brackets.

*** ,

** and

* mean significant at 1%, 5% and 10% levels respectively

### 4.2. Robustness test

#### 4.2.1. Change the measurement method.

In this paper, we further make the robustness test: First, we test the replacement estimation method. Using probit re-estimation (see Table 5), it can be found that, adding control variables in turn, The significance of the impact of social capital on youth migrant entrepreneurship and opportunistic entrepreneurship is consistent with the benchmark results. Specifically, the impact of social network, social status and social trust is still positive and significant. However, the effect of social participation is still not obvious, which is consistent with the above results. Furthermore, it is also found that social capital has a significant impact on opportunistic entrepreneurship, but not on survival entrepreneurship. The robustness of the benchmark results is also demonstrated.

**Table 5:** The impact of social capital on the entrepreneurial decision-making of young migrants.

	(1)	(2)	(3)	(4)	(5)	(6)	(7)
	Entery	Entery	Entery	Entery	Entery	Oppty	Surv
SC	0.028^***^					0.015^***^	0.006
	(0.004)					(0.002)	(0.004)
SI		0.020^***^					
		(0.002)					
ST			0.011^***^				
			(0.002)				
SEI				0.258^***^			
				(0.011)			
SA					0.007		
					(0.007)		
Personal characteristics	Yes	Yes	Yes	Yes	Yes	Yes	Yes
Family characteristics	Yes	Yes	Yes	Yes	Yes	Yes	Yes
Flow characteristics	Yes	Yes	Yes	Yes	Yes	Yes	Yes
Pro	Yes	Yes	Yes	Yes	Yes	Yes	Yes
N	38808	38808	38808	38808	38808	38808	38808

Note: Standard errors are in brackets.

*** ,

** and

* mean significant at 1%, 5% and 10% levels respectively

#### 4.2.2. Replace the core explanatory variables.

Second, replace the core explanatory variables. Select “Who do you associate most with locally in your spare time” [36], where you assign a value of one to the main association with other locals and a value of zero to the rest, Characterized by SC_1. Communication with local people means that the social network and social capital of young migrants are more concentrated and larger in scale. The regression results are shown in Table 6. Indicating that replacing the core explanatory variable, The impact of social capital on entrepreneurship and opportunistic entrepreneurship of young migrants is still significantly positive at the level of 1%. Consistent with the previous benchmark regression results.

**Table 6:** Effect of replacement of core explanatory variables.

	(1)	(2)	(3)
	Entery	Oppty	Surv
SC_1	0.014^***^	0.010^***^	0.004
	(0.005)	(0.002)	(0.005)
Personal characteristics	Yes	Yes	Yes
Family characteristics	Yes	Yes	Yes
Flow characteristics	Yes	Yes	Yes
Pro	Yes	Yes	Yes
N	38808	38808	38808

Note: Standard errors are in brackets.

*** ,

** and

* mean significant at 1%, 5% and 10% levels respectively

### 4.3. Heterogeneity analysis

In order to further explore the impact of social capital on the entrepreneurship of new generation of mobile youth, This part is further studied from the perspectives of different education levels, industries and regions.

#### 4.3.1. Heterogeneity of education levels.

Entrepreneurship is a systematic project including many links, such as opportunity mining, resource integration, enterprise creation and operation. Human capital has a significant impact on entrepreneurial behavior. In this paper, the floating population is divided into two groups of high education level and low education level according to whether the education level is university or above [37]. Table 7 reports the impact of social capital on the entrepreneurship of young migrant population groups with different education levels. The results in columns (1)-(2) show that Social capital plays a more important role in promoting the entrepreneurship of young migrants with lower education than those with higher education. But we found that for opportunistic entrepreneurship, The influence of social capital is more significant in the highly educated population (see columns (3)-(4) of Table 7). Wang et al. believe that migrants with higher education level are more likely to engage in wage work rather than self-employment [38]. The empirical results of this paper also show that in the high human capital group, the influence of social capital on entrepreneurship itself is relatively small. However, from the point of view of the type of entrepreneurship, there are more opportunities for entrepreneurship that need more resources, have greater risks and have more benefits. Social capital has a stronger impact on high human capital groups. To some extent, this also shows that the higher human capital group is more willing to try the entrepreneurial model with risks and benefits. Strong social capital will effectively promote their opportunistic entrepreneurship.

**Table 7:** Estimation of the Impact of Social Capital on Entrepreneurship of Samples with Different Education Levels.

	(1)	(2)	(3)	(4)	(5)	(6)
	High level of education	Low level of education	High level of education	Low level of education	High level of education	Low level of education
	Entery	Entery	Oppty	Oppty	Surv	Surv
SC	0.010	0.029^***^	0.012^***^	0.014^***^	−0.005	0.006
	(0.007)	(0.004)	(0.004)	(0.002)	(0.006)	(0.004)
Personal characteristics	Yes	Yes	Yes	Yes	Yes	Yes
Family characteristics	Yes	Yes	Yes	Yes	Yes	Yes
Flow characteristics	Yes	Yes	Yes	Yes	Yes	Yes
Pro	Yes	Yes	Yes	Yes	Yes	Yes
N	4432	34376	4406	34376	4432	34376

Note: Standard errors are in brackets.

*** ,

** and

* mean significant at 1%, 5% and 10% levels respectively

#### 4.3.2. Heterogeneity of family income.

Further, the sample is divided into high-income and low-income groups, with the high-income group indicating that the household income is above the average, On the contrary, it is the low-income group. This paper discusses the impact of social capital on the entrepreneurship of young and middle-aged migrants in different family income samples. The specific results are shown in Table 8. It is not difficult to find that both entrepreneurship and opportunistic entrepreneurship are significantly positive. Specifically, social capital in both groups increases the probability of entrepreneurship of young migrants, and the high-income group has a higher probability of entrepreneurship. Similarly, in the two groups, social capital plays a significant role in opportunistic entrepreneurship, and the high-income group plays a greater role in promoting entrepreneurship. For survival entrepreneurship, the impact of the two groups is not obvious.

**Table 8:** Estimation of the impact of social capital on entrepreneurship in different household income samples.

	(1)	(2)	(3)	(4)	(5)	(6)
	High-income group	Low-income group	High-income group	Low-income group	High-income group	Low-income group
	Entery	Entery	Oppty	Oppty	Surv	Surv
SC	0.040^***^	0.013^**^	0.028^***^	0.005^**^	0.003	0.008
	(0.006)	(0.005)	(0.003)	(0.002)	(0.006)	(0.005)
Personal characteristics	Yes	Yes	Yes	Yes	Yes	Yes
Family characteristics	Yes	Yes	Yes	Yes	Yes	Yes
Flow characteristics	Yes	Yes	Yes	Yes	Yes	Yes
Pro	Yes	Yes	Yes	Yes	Yes	Yes
N	1.5e + 04	2.4e + 04	1.5e + 04	2.4e + 04	1.5e + 04	2.4e + 04

Note: Standard errors are in brackets.

*** ,

** and

* mean significant at 1%, 5% and 10% levels respectively

#### 4.3.3. Industry heterogeneity.

Service industry, especially modern service industry, is the main driving force of China’s economic growth, and also an important area of youth entrepreneurship. According to the national economic industry classification standard, this paper divides the industry into two categories: service industry and non-service industry. On this basis, the service industry is further divided into modern service industry and traditional service industry [39]. To examine the impact of social capital on the entrepreneurship of young migrants in different industries, the estimated results are shown in Table 9. Columns (1)-(2) of Table 9 show that social capital has a significant impact on the entrepreneurship of young migrants in both service and non-service industries. However, the impact of social capital on the probability of entrepreneurship in modern service industry is significantly greater than that in traditional service industry (see columns (3)-(4) in Table 9). And we also found that, The impact of social capital on opportunistic entrepreneurship in modern service industry is more significant (see columns (5)-(8) in Table 9). Modern service industries such as finance, information services, business services, scientific research and technology are knowledge-intensive, technology-intensive, information-intensive and talent-intensive. Dense features, especially in the tide of digital economy, With the help of big data, cloud computing, Internet of Things and other information technologies, it has injected vitality and vitality into the modern service industry. Young people tend to have a strong ability to accept new things and are more sensitive to find new opportunities in modern service industries. For the modern service industry which focuses more on information and knowledge, social capital can effectively promote the flow of information. Reducing the entrepreneurial risk and financing constraints of the young floating population is conducive to the entrepreneurship of the young floating population. In particular, it is an opportunistic entrepreneurship with higher risks and greater benefits.

**Table 9:** Estimation of the impact of social capital on entrepreneurship in different industry samples.

	(1)	(2)	(3)	(4)	(5)	(6)	(7)	(8)
	Service industry	Non-service industry	Modern Service industry	Tradition Service industry	Modern Service industry	Tradition Service industry	Modern Service industry	Tradition Service industry
	Entery	Entery	Entery	Entery	Oppty	Oppty	Surv	Surv
SC	0.031^***^	0.023^***^	0.027^***^	0.032^***^	0.017^***^	0.014^***^	0.006	0.008
	(0.005)	(0.005)	(0.007)	(0.006)	(0.004)	(0.003)	(0.007)	(0.006)
Personal characteristics	Yes	Yes	Yes	Yes	Yes	Yes	Yes	Yes
Family characteristics	Yes	Yes	Yes	Yes	Yes	Yes	Yes	Yes
Flow characteristics	Yes	Yes	Yes	Yes	Yes	Yes	Yes	Yes
Pro	Yes	Yes	Yes	Yes	Yes	Yes	Yes	Yes
N	24661	14147	3085	21576	2804	21576	3085	24661

Note: Standard errors are in brackets.

*** ,

** and

* mean significant at 1%, 5% and 10% levels respectively

#### 4.3.4. Regional heterogeneity.

Social capital may also have a heterogeneous impact on the young migrant population in cities with different levels of development. Municipalities directly under the Central Government, sub-provincial cities and provincial capitals are the centers of floating population agglomeration, which are in line with the theoretical “central” cities. Therefore, it is divided into “central” cities, while the rest are “peripheral” cities [40]. In this paper, the sample is divided into “center-periphery” cities, and the results are shown in Table 10. Social capital has a significant impact on entrepreneurship in both central and peripheral cities. The impact on entrepreneurship in central cities is significantly greater than that in peripheral cities (see columns (1)-(2) of Panel A in Table 10). Further, we find that for opportunistic entrepreneurship, The impact of social capital on central cities is significantly greater than that on peripheral cities (see columns (3)-(4) of Panel A in Table 10). Central cities are the centers of regional economy, with good conditions for economic development and strong economies of scale and agglomeration. It has a strong attraction for the young floating population. In the central city, social capital can effectively integrate various resources to start a business for the young floating population. In particular, opportunity-based entrepreneurship provides better conditions. For peripheral cities, there are relatively few resources in the market, and social capital has little impact on the entrepreneurship of young migrants.

**Table 10:** Estimation of the impact of social capital on entrepreneurship in different regions.

Panel A	Center- Periphery sampled					
	(1)	(2)	(3)	(4)	(5)	(6)
	Central cities	Periphery cities	Central cities	Periphery cities	Central cities	Periphery cities
	Entery	Entery	Oppty	Oppty	Surv	Surv
SC	0.037^***^	0.022^***^	0.015^***^	0.012^***^	0.013^**^	0.003
	(0.005)	(0.006)	(0.002)	(0.003)	(0.005)	(0.006)
Personal characteristics	Yes	Yes	Yes	Yes	Yes	Yes
Family characteristics	Yes	Yes	Yes	Yes	Yes	Yes
Flow characteristics	Yes	Yes	Yes	Yes	Yes	Yes
Pro	Yes	Yes	Yes	Yes	Yes	Yes
N	21280	17528	21280	17528	21280	17528
**Panel B**	Coastal-Inland sampled					
	(1)	(2)	(3)	(4)	(5)	(6)
	Coastal areas	Inland areas	Coastal areas	Inland areas	Coastal areas	Inland areas
	Entery	Entery	Oppty	Oppty	Surv	Surv
SC	0.028^***^	0.028^***^	0.013^***^	0.015^***^	0.008	0.005
	(0.006)	(0.005)	(0.002)	(0.002)	(0.006)	(0.005)
Personal characteristics	Yes	Yes	Yes	Yes	Yes	Yes
Family characteristics	Yes	Yes	Yes	Yes	Yes	Yes
Flow characteristics	Yes	Yes	Yes	Yes	Yes	Yes
Pro	Yes	Yes	Yes	Yes	Yes	Yes
N	18427	20381	18427	20381	18427	20381

Note: Standard errors are in brackets.

*** ,

** and

* mean significant at 1%, 5% and 10% levels respectively

According to the Classification and Code of Coastal Administrative Region (HY/T094-2006), the sample is divided into coastal areas and inland areas (see Table 10 Panel B). The estimation results show that social capital in both coastal and inland areas has a significant impact on entrepreneurship and opportunistic entrepreneurship. However, the impact of social capital on opportunistic entrepreneurship in coastal areas is stronger. China’s reform and opening up has shown the characteristics of regional imbalance, and the coastal areas often have better economic development conditions. The market development is relatively perfect, which also creates more opportunities for entrepreneurs and attracts a large number of young workers. For these areas, social capital can better integrate relevant resources, which is conducive to entrepreneurship, especially opportunistic entrepreneurship. The entrepreneurship of young migrants in these areas has further stimulated their market vitality and injected vitality into the development of coastal areas. Social capital is under great pressure for young migrants to start their own businesses in different cities. It may also further bring about the “polarization effect” of central cities and coastal cities, and strengthen the “Matthew effect” of regional development.

## 5. Expanded analysis

### 5.1. Moderating effects modeling

In order to explore the moderating effect of digital economy on the impact of social capital on youth entrepreneurship, the following model is constructed to test its moderating effect. The specific form is as follows:



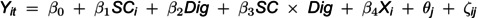

(2)


Among them, Dig is the regulating variable, which represents the digital economy. SC × Dig is the interaction term between social capital and digital economy, and here we mainly focus on the sign of the coefficient β3 of the interaction term SC × Dig. The interaction coefficient can indicate whether the moderating variables enhance or weaken the impact of the digital economy on youth entrepreneurship. The setting of the remaining variables is the same as in Equation (1).

### 5.2. Selection of indicators for moderating variables

The moderating variable is digital economy. Referring to the related study of Zhao Tao et al. [40], the digital economy is measured by the guest entropy method using the indicators of five dimensions, namely the number of Internet users per 100 people in prefecture-level cities, the percentage of employees in the computer services and software industry, the total telecommunication business per capita and the total postal business per capita, the number of cell phone subscribers per 100 people and the digital finance. The city-level digital economy is further matched with CMDS data to obtain the final digital economy indicator, denoted as Dig.

### 5.3. Selection of indicators for moderating variables

Table 11 shows the regression results of the moderating effect model. The empirical results show that the cross-product coefficient of social capital (SC) and digital economy (Dig1) is significantly positive. It shows that the digital economy positively regulates the role of social capital in promoting youth entrepreneurship, and the promotion effect of social capital on youth entrepreneurship has also increased. The above empirical results support Hypothesis 3. The possible reason for this is that, in the face of the impact of the digital economy, social capital plays a protective role for entrepreneurship and increases the risk-resistant capacity of entrepreneurs. The digital economy further breaks down the original barriers to social capital, broadens the channels through which entrepreneurs can acquire knowledge, skills and experience, enhances the convenience and timeliness of exchanges and communication, accelerates the flow of entrepreneurial factors, and helps to raise the level of entrepreneurs’ social capital, which in turn promotes the growth of entrepreneurship rates.

**Table 11:** Moderating effect test.

	(1)
	Entery
SC	0.022^***^
	(0.005)
Dig	−0.488^***^
	(0.018)
SC_Dig	0.013^*^
	(0.007)
Personal characteristics	Yes
Family characteristics	Yes
Flow characteristics	Yes
Pro	Yes
N	38808

Note: Standard errors are in brackets.

*** ,

** and

* mean significant at 1%, 5% and 10% levels respectively.

## 6. Research conclusions and policy implications

### 6.1. Research conclusion

Based on the dynamic monitoring data of the floating population, this paper empirically analyzes the impact of social capital on the entrepreneurship of the young floating population. The conclusions are as follows: (1) Social capital has significantly increased the participation of young migrants in entrepreneurship. Its impact on opportunity entrepreneurship is significantly greater than that on survival entrepreneurship.(2) Social network, social trust and social status can significantly promote the entrepreneurship of young migrants. And the impact on opportunity entrepreneurship is greater than that on survival entrepreneurship, while the impact of social participation on entrepreneurship is not significant.(3) Social capital has a greater impact on entrepreneurship in groups with lower education level. However, it has a greater impact on group opportunistic entrepreneurship with higher education level; Compared with the group with low family income, social capital has a greater impact on the opportunistic entrepreneurship of the group with high family income; Compared with traditional service industry and non-service industry, social capital has a greater impact on opportunistic entrepreneurship in modern service industry. Compared with peripheral cities and inland areas, Social capital has a more significant impact on the opportunistic entrepreneurship of young migrants in central cities and coastal areas.(4) Digital economy plays a positive regulatory role in social capital promoting the entrepreneurship of the new generation of migrant youth.

### 6.2. Policy implications

The above conclusions give us some enlightenment:

First, we should further improve the formation of social capital of young floating population and create a good environment for digital entrepreneurship. This empirical study shows that social capital is an important driving force to promote the entrepreneurship of young migrants. In order to continue to promote the development of digital technology in the future, the government should build a network platform for digital entrepreneurship. It provides necessary support from financial subsidies and financial support. We will further expand the social network relationship of the young floating population based on their professional ties and lower the threshold for their integration into the local society. Improve social trust. Government agencies and industry associations should also actively establish a humanized and functional platform for digital innovation and entrepreneurship. Form the gathering of talents, further improve social capital, and better promote opportunistic entrepreneurship. The government should also provide preferential policies for migrant youth to start their own businesses, create a platform for digital entrepreneurship, reduce their start-up costs and stimulate their entrepreneurial vitality.

Secondly, we should give full play to the advantages of the Communist Youth League and enhance the role of social participation in the entrepreneurship of young migrants. From the empirical study, we find that social participation, as an important dimension of social capital, has limited impact on entrepreneurship. In the future, the Communist Youth League should play a greater role in the formation of social capital of young floating population. Through more targeted digital innovation and entrepreneurship training courses for young migrants, the establishment of digital innovation and entrepreneurship bases for young people, Youth Digital Innovation and Entrepreneurship Competition and other measures, From the perspective of service entrepreneurship, we will further expand the online and offline social participation of young migrants. By improving the social participation of young migrants, the understanding and trust among entrepreneurs will be continuously enhanced. To form a network of interpersonal relationships with common value orientation and high trust, so as to provide better conditions for young migrants to start their own businesses.

Thirdly, we should focus on improving the synergy between human capital and social capital in the entrepreneurship of young labor force. From the empirical results of this paper, it is found that people with higher education human capital, Social capital plays a more obvious role in promoting opportunistic entrepreneurship. In the future, we should further strengthen the promotion of the cultural level of the young floating population as an important policy grasp. On the one hand, we should pay attention to improving the cultural level of the young floating population, especially the cultural level of the lower human capital groups; On the other hand, we should pay attention to improving and expanding the existing vocational training system, and increase the number of digital and intelligent related specialties and curriculum settings. Enhance the ability of labor force to adapt to the digital economy, especially to create better conditions for social capital to better promote opportunistic entrepreneurship. In addition, a digital technology entrepreneurship platform should be set up to provide young entrepreneurs with good communication channels and broaden their social relations.

Fourthly, central cities and coastal cities tend to have a higher level of economic development and a better market environment, which can be used for entrepreneurship. In particular, it provides a better platform for opportunistic entrepreneurship. In the future, on the one hand, we should constantly improve the entrepreneurial environment of central cities and coastal cities, and pay attention to promoting digital cooperation between cities. We should give full play to the leading role of developed regions and provide support for social capital to promote a higher level of entrepreneurship. On the other hand, we should also pay attention to the benign interaction between the central and peripheral cities, coastal and inland areas, and the development of digital technology. We should cultivate social capital in peripheral cities and inland cities, and try our best to overcome the “Matthew effect” of inter-regional entrepreneurship. Narrowing the “digital divide”.

## Supporting information

S1 File Data. (XLS)

S2 File Dofile-submit. (DOCX)
