# Supplementary material for: Social capital, digital economy and the entrepreneurship of the new generation of migrant youth: Empirical analysis based on CMDS data
Source: PLoS One. 2025 Jun 25;20(6):e0322458. doi: 10.1371/journal.pone.0322458 (PMC12194044; doi:10.1371/journal.pone.0322458)
Supplement: S2 File — (DOCX) [file pone.0322458.s002.docx]

sum SC age age_sq gender marry edu1 nation health health_record house politic hukou

**Benchmark regression**

logit entery SC age age_sq gender marry edu1 nation health health_record house politic hukou i.province , nolog

margins, dydx(*) post

est store m1

logit entery SC age age_sq gender marry edu1 nation health health_record house politic hukou lnfamily_income familysize i.province, nolog

margins, dydx(*) post

est store m2

logit entery SC age age_sq gender marry edu1 nation health health_record house politic hukou lnfamily_income familysize city_num flow_range i.province, nolog

margins, dydx(*) post

est store m3

logit entery SI age age_sq gender marry edu1 nation health health_record house politic hukou lnfamily_income familysize city_num flow_range i.province, nolog

margins, dydx(*) post

est store m4

logit entery ST age age_sq gender marry nation edu1 health health_record house politic hukou lnfamily_income familysize city_num flow_range i.province, nolog

margins, dydx(*) post

est store m5

logit entery SEI age age_sq gender marry nation edu1 health health_record house politic hukou lnfamily_income familysize city_num flow_range i.province, nolog

margins, dydx(*) post

est store m6

logit entery SA age age_sq gender marry nation edu1 health health_record house politic hukou lnfamily_income familysize city_num flow_range i.province, nolog

margins, dydx(*) post

est store m7

**oppty

logit oppty SC age age_sq gender marry nation edu1 health health_record house politic hukou i.province, nolog

margins, dydx(*) post

est store m1

logit oppty SC age age_sq gender marry nation edu1 health health_record house politic hukou lnfamily_income familysize i.province, nolog

margins, dydx(*) post

est store m2

logit oppty SC age age_sq gender marry nation edu1 health health_record house politic hukou lnfamily_income familysize city_num flow_range i.province, nolog

margins, dydx(*) post

est store m3

**surv

logit surv SC age age_sq gender marry nation edu1 health health_record house politic hukou i.province, nolog

margins, dydx(*) post

est store m4

logit surv SC age age_sq gender marry nation edu1 health health_record house politic hukou lnfamily_income familysize i.province, nolog

margins, dydx(*) post

est store m5

logit surv SC age age_sq gender marry nation edu1 health health_record house politic hukou lnfamily_income familysize city_num flow_range i.province, nolog

margins, dydx(*) post

est store m6

**type**

**oppty

logit oppty SI age age_sq gender marry nation edu1 health health_record house politic hukou lnfamily_income familysize city_num flow_range i.province, nolog

margins, dydx(*) post

est store m1

logit oppty ST age age_sq gender marry nation edu1 health health_record house politic hukou lnfamily_income familysize city_num flow_range i.province, nolog

margins, dydx(*) post

est store m2

logit oppty SEI age age_sq gender marry nation edu1 health health_record house politic hukou lnfamily_income familysize city_num flow_range i.province, nolog

margins, dydx(*) post

est store m3

logit oppty SA age age_sq gender marry nation edu1 health health_record house politic hukou lnfamily_income familysize city_num flow_range i.province, nolog

margins, dydx(*) post

est store m4

**surv

logit surv SI age age_sq gender marry nation edu1 health health_record house politic hukou lnfamily_income familysize city_num flow_range i.province, nolog

margins, dydx(*) post

est store m5

logit surv ST age age_sq gender marry nation edu1 health health_record house politic hukou lnfamily_income familysize city_num flow_range i.province, nolog

margins, dydx(*) post

est store m6

logit surv SEI age age_sq gender marry nation edu1 health health_record house politic hukou lnfamily_income familysize city_num flow_range i.province, nolog

margins, dydx(*) post

est store m7

logit surv SA age age_sq gender marry nation edu1 health health_record house politic hukou lnfamily_income familysize city_num flow_range i.province, nolog

margins, dydx(*) post

est store m8

*================================

***********robust***********

*================================

probit entery SC age age_sq gender marry nation edu1 health health_record house politic hukou lnfamily_income familysize city_num flow_range i.province, nolog

margins,dydx(*) post

est store m1

probit entery SI age age_sq gender marry nation edu1 health health_record house politic hukou lnfamily_income familysize city_num flow_range i.province, nolog

margins,dydx(*) post

est store m2

probit entery ST age age_sq gender marry nation edu1 health health_record house politic hukou lnfamily_income familysize city_num flow_range i.province, nolog

margins,dydx(*) post

est store m3

probit entery SEI age age_sq gender marry nation edu1 health health_record house politic hukou lnfamily_income familysize city_num flow_range i.province, nolog

margins,dydx(*) post

est store m4

probit entery SA age age_sq gender marry nation edu1 health health_record house politic hukou lnfamily_income familysize city_num flow_range i.province, nolog

margins,dydx(*)

est store m5

probit oppty SC age age_sq gender marry nation edu1 health health_record house politic hukou lnfamily_income familysize city_num flow_range i.province, nolog

margins,dydx(*) post

est store m6

probit surv SC age age_sq gender marry nation edu1 health health_record house politic hukou lnfamily_income familysize city_num flow_range i.province, nolog

margins,dydx(*) post

est store m7

logit entery contact age age_sq gender marry nation edu1 health health_record house politic hukou lnfamily_income familysize city_num flow_range i.province, nolog

margins,dydx(*) post

est store m1

logit oppty contact age age_sq gender marry nation edu1 health health_record house politic hukou lnfamily_income familysize city_num flow_range i.province, nolog

margins,dydx(*) post

est store m2

logit surv contact age age_sq gender marry nation edu1 health health_record house politic hukou lnfamily_income familysize city_num flow_range i.province, nolog

margins,dydx(*) post

est store m3

*=====================================================

**********heterogeneity regression**********

*=====================================================

****edu2****

sum SC if edu2==1

sum SC if edu2==0

sum SC if f_inc_l==1

sum SC if f_inc_l==0

sum SC if ind==1

sum SC if ind==0

sum SC if ind_hl==1

sum SC if ind_hl==0

sum SC if coast==1

sum SC if coast==0

sum SC if center==1

sum SC if center==0

logit entery SC age age_sq gender marry edu1 nation health health_record house politic hukou lnfamily_income familysize city_num flow_range i.province if edu2==1, nolog

margins, dydx(*) post

est store m1

logit entery SC age age_sq gender marry edu1 nation health health_record house politic hukou lnfamily_income familysize city_num flow_range i.province if edu2==0, nolog

margins, dydx(*) post

est store m2

logit oppty SC age age_sq gender marry edu1 nation health health_record house politic hukou lnfamily_income familysize city_num flow_range i.province if edu2==1, nolog

margins, dydx(*) post

est store m3

logit oppty SC age age_sq gender marry edu1 nation health health_record house politic hukou lnfamily_income familysize city_num flow_range i.province if edu2==0, nolog

margins, dydx(*) post

est store m4

logit surv SC age age_sq gender marry edu1 nation health health_record house politic hukou lnfamily_income familysize city_num flow_range i.province if edu2==1, nolog

margins, dydx(*) post

est store m5

logit surv SC age age_sq gender marry edu1 nation health health_record house politic hukou lnfamily_income familysize city_num flow_range i.province if edu2==0, nolog

margins, dydx(*) post

est store m6

****f_inc_l****

logit entery SC age age_sq gender marry nation edu1 health health_record house politic hukou lnfamily_income familysize city_num flow_range i.province if f_inc_l==1, nolog

margins, dydx(*) post

est store m1

logit entery SC age age_sq gender marry nation edu1 health health_record house politic hukou lnfamily_income familysize city_num flow_range i.province if f_inc_l==0, nolog

margins, dydx(*) post

est store m2

logit oppty SC age age_sq gender marry nation edu1 health health_record house politic hukou lnfamily_income familysize city_num flow_range i.province if f_inc_l==1, nolog

margins, dydx(*) post

est store m3

logit oppty SC age age_sq gender marry nation edu1 health health_record house politic hukou lnfamily_income familysize city_num flow_range i.province if f_inc_l==0, nolog

margins, dydx(*) post

est store m4

logit surv SC age age_sq gender marry nation edu1 health health_record house politic hukou lnfamily_income familysize city_num flow_range i.province if f_inc_l==1, nolog

margins, dydx(*) post

est store m5

logit surv SC age age_sq gender marry nation edu1 health health_record house politic hukou lnfamily_income familysize city_num flow_range i.province if f_inc_l==0, nolog

margins, dydx(*) post

est store m6

****ind****

logit entery SC age age_sq gender marry edu1 nation health health_record house politic hukou lnfamily_income familysize city_num flow_range i.province if ind==1, nolog

margins, dydx(*) post

est store m1

logit entery SC age age_sq gender marry edu1 nation health health_record house politic hukou lnfamily_income familysize city_num flow_range i.province if ind==0, nolog

margins, dydx(*) post

est store m2

logit entery SC age age_sq gender marry edu1 nation health health_record house politic hukou lnfamily_income familysize city_num flow_range i.province if ind_hl==1, nolog

margins, dydx(*) post

est store m3

logit entery SC age age_sq gender marry edu1 nation health health_record house politic hukou lnfamily_income familysize city_num flow_range i.province if ind_hl==0, nolog

margins, dydx(*) post

est store m4

logit oppty SC age age_sq gender marry edu1 nation health health_record house politic hukou lnfamily_income familysize city_num flow_range i.province if ind_hl==1, nolog

margins, dydx(*) post

est store m5

logit oppty SC age age_sq gender marry edu1 nation health health_record house politic hukou lnfamily_income familysize city_num flow_range i.province if ind_hl==0, nolog

margins, dydx(*) post

est store m6

logit surv SC age age_sq gender marry edu1 nation health health_record house politic hukou lnfamily_income familysize city_num flow_range i.province if ind_hl==1, nolog

margins, dydx(*) post

est store m7

logit surv SC age age_sq gender marry edu1 nation health health_record house politic hukou lnfamily_income familysize city_num flow_range i.province if ind_hl==0, nolog

margins, dydx(*) post

est store m8

****center****

logit entery SC age age_sq gender marry nation edu1 health health_record house politic hukou lnfamily_income familysize city_num flow_range i.province if center==1, nolog

margins, dydx(*) post

est store m1

logit entery SC age age_sq gender marry nation edu1 health health_record house politic hukou lnfamily_income familysize city_num flow_range i.province if center==0, nolog

margins, dydx(*) post

est store m2

logit oppty SC age age_sq gender marry nation edu1 health health_record house politic hukou lnfamily_income familysize city_num flow_range i.province if center==1, nolog

margins, dydx(*) post

est store m3

logit oppty SC age age_sq gender marry nation edu1 health health_record house politic hukou lnfamily_income familysize city_num flow_range i.province if center==0, nolog

margins, dydx(*) post

est store m4

logit surv SC age age_sq gender marry nation edu1 health health_record house politic hukou lnfamily_income familysize city_num flow_range i.province if center==1, nolog

margins, dydx(*) post

est store m5

logit surv SC age age_sq gender marry nation edu1 health health_record house politic hukou lnfamily_income familysize city_num flow_range i.province if center==0, nolog

margins, dydx(*) post

est store m6

****coast****

logit entery SC age age_sq gender marry nation edu1 health health_record house politic hukou lnfamily_income familysize city_num flow_range i.province if coast==1, nolog

margins, dydx(*) post

est store m1

logit entery SC age age_sq gender marry nation edu1 health health_record house politic hukou lnfamily_income familysize city_num flow_range i.province if coast==0, nolog

margins, dydx(*) post

est store m2

logit oppty SC age age_sq gender marry nation edu1 health health_record house politic hukou lnfamily_income familysize city_num flow_range i.province if coast==1, nolog

margins, dydx(*) post

est store m3

logit oppty SC age age_sq gender marry nation edu1 health health_record house politic hukou lnfamily_income familysize city_num flow_range i.province if coast==0, nolog

margins, dydx(*) post

est store m4

logit surv SC age age_sq gender marry nation edu1 health health_record house politic hukou lnfamily_income familysize city_num flow_range i.province if coast==1, nolog

margins, dydx(*) post

est store m5

logit surv SC age age_sq gender marry nation edu1 health health_record house politic hukou lnfamily_income familysize city_num flow_range i.province if coast==0, nolog

margins, dydx(*) post

est store m6

*Expand

logit entery SC Dig sc_dig age age_sq gender marry edu1 nation health health_record house politic hukou lnfamily_income familysize city_num flow_range i.province, nolog

margins, dydx(*) post

est store m1
